# Neurovascular phase coherence is altered in Alzheimer’s disease

**DOI:** 10.1093/braincomms/fcaf007

**Published:** 2025-02-03

**Authors:** Juliane Bjerkan, Bernard Meglič, Gemma Lancaster, Jan Kobal, Peter V E McClintock, Trevor J Crawford, Aneta Stefanovska

**Affiliations:** Department of Physics, Lancaster University, LA1 4YB Lancaster, UK; Department of Neurology, University Medical Centre, 1525 Ljubljana, Slovenia; Department of Physics, Lancaster University, LA1 4YB Lancaster, UK; Department of Neurology, University Medical Centre, 1525 Ljubljana, Slovenia; Department of Physics, Lancaster University, LA1 4YB Lancaster, UK; Department of Psychology, Lancaster University, LA1 4YF Lancaster, UK; Department of Physics, Lancaster University, LA1 4YB Lancaster, UK

**Keywords:** neurovascular unit, time-frequency analysis, multi-scale oscillatory analysis, phase coherence, brain oxygenation

## Abstract

Alzheimer’s disease is the commonest form of dementia, but its cause still remains elusive. It is characterized by neurodegeneration, with amyloid-beta and tau aggregation. Recently, however, the roles of the vasculature and the neurovascular unit are being highlighted as important for disease progression. In particular, there is reduced microvascular density, and altered gene expression in vascular and glial cells. Structural changes naturally impact the functioning of the neurovascular unit, and the goal of the study was to quantify the corresponding changes *in vivo*, non-invasively. Our assessment is based on recordings of brain oxygenation, neuronal and cardiorespiratory activities, captured by functional near-infrared spectroscopy, electroencephalogram, electrocardiogram and respiration effort, respectively. Two groups were compared: an Alzheimer’s disease group (N = 19) and a control group (N = 20) of similar age. The time-series were analysed using methods that can capture multi-scale and time-varying oscillations such as the wavelet transform power and wavelet phase coherence. The Alzheimer’s disease group shows a significant decrease in the power of brain oxygenation oscillations compared to the control group. There is also a significant global reduction in the phase coherence between brain oxygenation time-series. The neurovascular phase coherence around 0.1 Hz is also significantly reduced in the Alzheimer’s disease group. In addition, the average respiration rate is increased in the Alzheimer’s disease group compared to the control group. We show that the phase coherence between vascular and neuronal activities is reduced in Alzheimer’s disease compared to the control group, indicating altered functioning of the neurovascular unit. The brain oxygenation dynamics reveals reduced power and coordination of oscillations, especially in frequency ranges that are associated with vasomotion. This could lead to reduced oxygen delivery to the brain, which could affect ATP production, and potentially reduce amyloid-beta clearance. These changes in neurovascular dynamics have potential for early diagnosis, as a marker of disease progression, and for evaluating the effect of interventions.

## Teaser

Bjerkan et al. assessed the functioning of the neurovascular unit in Alzheimer’s disease through non-invasive measurements of brain electrical activity, brain oxygenation, heart rate and respiration. They find decreased neurovascular phase coherence and oxygenation power in Alzheimer’s disease patients compared to controls, but increased respiration rate in Alzheimer’s disease patients.

## Graphical Abstract

**Graphical Abstract:**
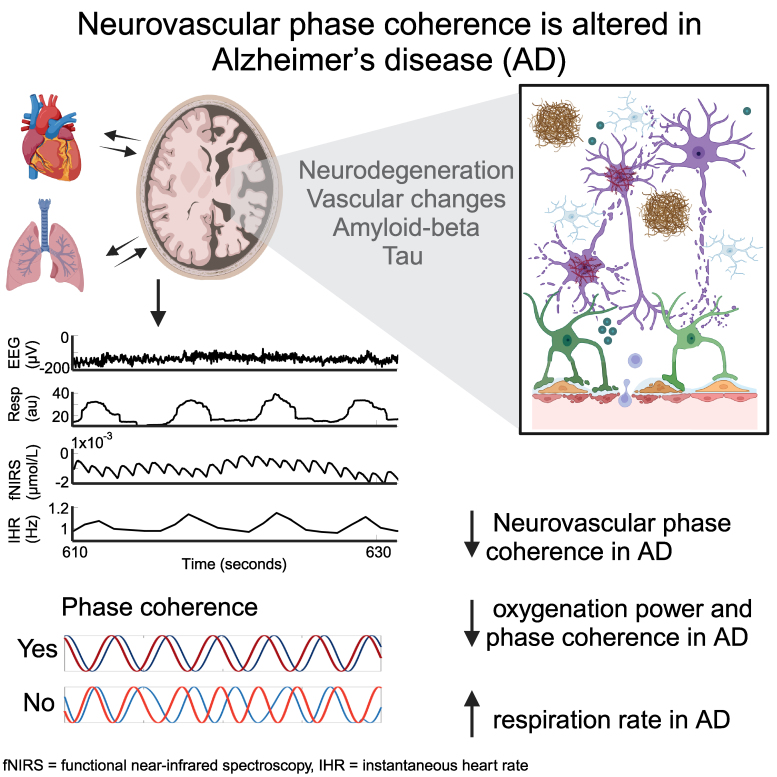

## Introduction

Alzheimer’s disease is a neurodegenerative disease leading to memory problems and a decline in cognitive function. It is the commonest form of dementia, and is especially prevalent in the older population. The mechanisms leading to Alzheimer’s disease are not fully elucidated, but Alzheimer’s disease is associated with amyloid-beta deposits,^1,2^ tau protein tangles,^3^ brain atrophy^4^ and vascular changes.^5^ Vascular and neurovascular pathways to neurodegeneration are increasingly being recognized as important for both the onset and progression of the disease.^6,7^ The two-hit vascular hypothesis singles out vascular factors, such as hypertension and diabetes, and genetic factors, such as APOE4, to initiate vascular damage (hit one).^8^ This then leads to reduced amyloid-beta clearance leading to amyloid-beta accumulation (hit two). According to the hypothesis the two hits, both independently and synergistically, cause synaptic dysfunction and neurodegeneration, which can be seen as disrupted structural and functional connectivity in the brain. This has a major effect on brain function, and leads to the symptoms of dementia.^8^

The brain consumes around 20% of the body’s energy usage, and works together with the cardiovascular system to maintain a balance between local energy demand and supply. This is ensured through neurovascular coupling, controlled by the neurovascular unit (NVU).^9^ The NVU consists of many cell types, such as astrocytes, vascular smooth muscle cells (VSMC), neurons and endothelial cells. There is evidence of neurovascular de-coupling in Alzheimer’s disease, such as dysregulation of cerebral blood flow, potentially caused by alterations in VSMCs and astrocytes.^8,10^ Interestingly, neurovascular de-coupling is implicated in cognitive decline in both ageing and Alzheimer’s disease,^11^ and several vascular and glial cells have genes that are expressed differently in Alzheimer’s disease compared to healthy controls.^12^ Hence, evaluating neurovascular interactions non-invasively in Alzheimer’s disease participants could provide useful insights into the disease progression and mechanisms, potentially aiding in diagnosis and the assessment of future treatments.

Earlier research found that electrical activity (measured by electroencephalography (EEG) or neuropixels probes) is correlated with hemodynamic activity (measured with functional near-infrared spectroscopy (fNIRS) or fMRI) at the same slow frequencies (around 0.1 Hz), both in the healthy human and mouse brains.^13-17^ We have reported previously that this correlation decreases with ageing,^17^ but no study has yet investigated such a relationship in participants with Alzheimer’s disease. We hypothesise that, due to neurovascular de-coupling, the neurovascular coherence will be reduced. To test this, we employed fNIRS and EEG. Both techniques have good temporal resolution,^18-20^ and are therefore well-suited to studies of time-varying phase dynamics. In addition, the methods are non-invasive and relatively inexpensive, making them an attractive option compared to methods such as fMRI and positron emission tomography (PET).

**Figure 1:**
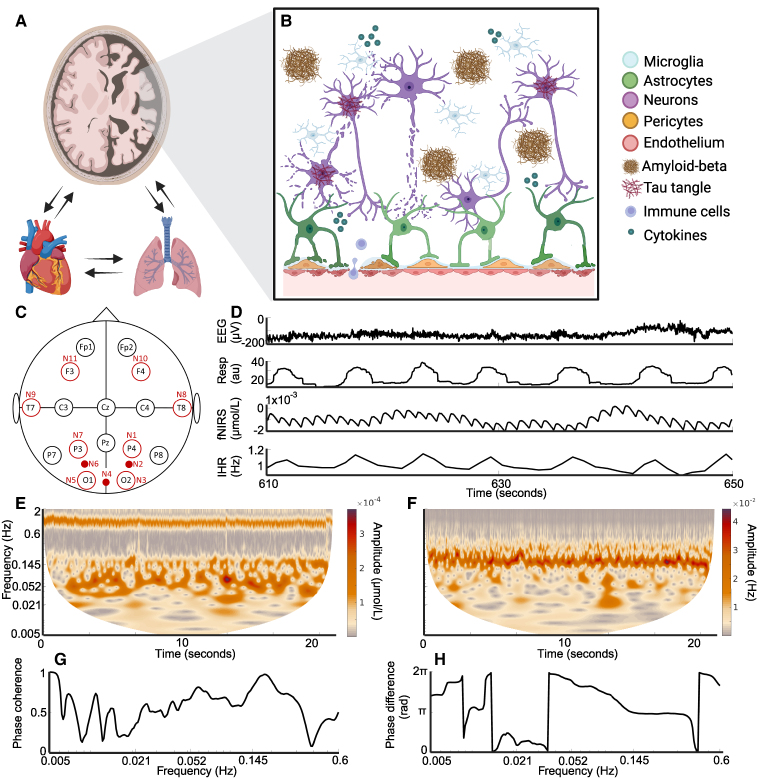
**Overview figure.** (**A**) Diagram of the brain, heart and lungs. Notice that the right side of the brain illustrates the atrophy associated with Alzheimer’s disease. The arrows indicate interactions between the systems. (**B**) Illustration of the NVU, showing how astrocytes link the blood vessels and neurons. Alzheimer’s disease is associated with a leaky blood–brain-barrier, inflammation, neurodegeneration, amyloid-beta plaques and tau tangles. (**C**) The probe layout for fNIRS and electroencephalogram (EEG). The open black circles indicate EEG probes, the open red circles indicate both an EEG and a fNIRS probe, while the small red circles indicate only fNIRS probes. (**D**) Example time-series measured from a control participant, shown for 30 s. IHR, instantaneous heart rate; Resp, respiration. (**E**) The WT of the fNIRS time-series from one participant. (**F**) The WT of the IHR time-series from the same participant. The *y*-axis is the same as in (**E**). (**G**) The WPC between the fNIRS and IHR time-series. (**H**) The phase difference between the fNIRS and IHR time-series. Rad, radians. Created in BioRender. Bjerkan, J. (2025) https://BioRender.com/z13u692.

We treat the cardiovascular system and the brain as systems of interacting oscillators,^21^ recognizing that living systems, being thermodynamically open systems and far-from-equilibrium, exhibit oscillations with time-varying frequencies.^22,23^ In view of the latter, methods able to capture multi-scale time-localized dynamics with logarithmic frequency resolution need to be used.^24^ Time-frequency analysis methods such as the wavelet transform (WT) and wavelet phase coherence (WPC)^25-27^ were accordingly applied to the measured time-series as illustrated in Fig. 1.

The brain does not work in isolation from the cardiovascular and respiratory systems, and hence we also recorded simultaneously the heart rate (from ECG) and respiration effort (from a respiration belt). Systemic cardiovascular oscillations naturally impact brain oxygenation, and so it is especially important to understand the oscillations in fNIRS.

The overall aim of the study was to evaluate quantitatively, we believe for the first time, how the cardiovascular and neurovascular phase interactions change in participants with Alzheimer’s disease. According to the two-hit vascular hypothesis either or both could play a role in the development of the disease. There is growing recognition that Alzheimer’s disease is a multi-system disorder and should be regarded as a non-linear dynamical disorder.^28^ Consideration of dynamics in health and disease calls for studies of oscillations.^29^ We therefore combine non-invasive measurements of cardiovascular and neurovascular dynamics, with state-of-the-art time-frequency analysis methods, in order to obtain a more integrated picture of Alzheimer’s disease.

## Materials and methods

### Participants

The study was conducted according to the Declaration of Helsinki. Study protocols were approved by the Commission of the Republic of Slovenia for Medical Ethics or by the Faculty of Science and Technology Research Ethics Committee (FSTREC) at Lancaster University. Written informed consent was given by all participants.

Measurements were conducted on 29 Alzheimer’s disease participants (18 F, 11 M). Out of these, six were excluded as they were older than the control participants, and therefore it was not possible to match the groups; two were excluded due to not completing the full recordings; one was excluded due to the participant touching the wires and cap multiple times, causing poor signal quality; one was excluded due to being an outlier (a combination of tachypnoea, atrial fibrillation and obesity). Data of included Alzheimer’s disease patients can be found in Table 1.

**Table 1:** Participant data and patient data

Participant data							
	N	Age (years)	Sex	BMI (kgm^−2^)	sBP (mmHg)	dBP (mmHg)	MMSE
AD	19	70.9 ± 6.7	12F/7M	25.2 ± 3.9	135 ± 23	79 ± 12	21.8 ± 4.9
C	20	67.8 ± 6.9	11F/9M	27.5 ± 3.0	139 ± 16	85 ± 11	
*P*		0.125		0.0543	0.173	0.140	

Patient data							
			CSF				
NN	Age (years)	Sex	P tau (pg/ml)	Tau (pg/ml)	Amyloid-beta 1–42 (pg/ml)	MMSE	Stage
1	56	F	107	704	580	24	1
2	68	M	63	398	417	NA	1
3	73	F	203	1568	397	19	1
4	77	F	382	67	557	24	1
5	68	M	146	29	431	28	0
6	60	F	1150	130	512	15	2
7	60	M	520	63	531	11	1
8	77	M	862	152	442	22	1
9	78	M	1403	134	545	18	2
10	76	F	885	108	572	17	2
11	69	F	2068	200	494	20	2
12	77	F	328	46	314	18	2
13	73	F	500	68	533	28	0
14	73	M	1347	221	373	25	1
15	63	F	1080	133	735	20	1
16	73	F	315	55	455	29	0
17	76	F	464	63	536	27	0
18	75	F	647	93	526	24	1
19	76	M	530	77	515	23	2

AD, Alzheimer’s disease group; C, control group; N, number (of participants); *P*, *P*-value from the Wilcoxon rank-sum test comparing the groups; BMI, body mass index; sBP, systolic blood pressure; dBP, diastolic blood pressure; NN, participant number; CSF, cerebral spinal fluid; M, male; F, female; P tau, phosphorylated tau; MMSE, mini mental state exam. All MMSE scores were obtained within 1.5 months of the recording, apart from the scores of Patients 3, 4, 7 and 15 (obtained within 3 months). Stage 0 corresponds to MCI, Stage 1 to mild disease and Stage 2 to moderate disease. The cut-off for normal levels are approximately^30,31^: P tau <400 pg/ml, tau <60 pg/ml and amyloid 1–42 > 570 pg/ml.

Alzheimer’s disease was diagnosed by clinical evaluation, and the presence of abnormal tau and/or amyloid-beta 1–42 concentrations in the cerebrospinal fluid^30,31^ (CSF) (Table 1). Four people in the Alzheimer’s disease group were mild cognitive impairment (MCI) patients, and had CSF amyloid-beta or tau levels which strongly suggested Alzheimer’s disease.

Twenty control participants of similar ages to the Alzheimer’s disease participants were also included. Exclusion criteria for the control group were not completing the full recordings, movement artefacts causing poor signal quality or having a BMI ≥ 40. Participants with Class 3 obesity were excluded to better align the BMI of the control and Alzheimer’s disease groups.

Data were analysed from a total of 39 participants: an Alzheimer’s disease group (N = 19) and a control group (N = 20). All participants were from Slovenia, apart from seven of the control participants who were from England.

In analyses involving respiration, six control participants were excluded. For five of them, the respiration belt had not been placed correctly, and therefore failed to measure the respiration. For the sixth participant, respiration was not measured.

In analyses involving instantaneous heart rate (IHR), six participants (two control and four Alzheimer’s disease) were excluded. Four participants (one control and three Alzheimer’s disease) were excluded due to abnormal ECGs, and one further Alzheimer’s disease participant was excluded due to a noisy ECG signal. For the sixth participant, ECG was not measured. The four abnormal ECGs are shown in the Supplementary material section 2.

Based on the group sizes, a power of 0.8 and an alpha of 0.05, the effect size sensitivity analysis performed in G*Power reveal that an effect size of 1.0 or more can reliably be detected in this study.

### Data acquisition

During the measurement, each participant was sitting in a comfortable chair in a quiet room at the Neurology Clinic, University Medical Center Ljubljana, Ljubljana, Slovenia or in the Nonlinear and Biomedical Physics Lab, Lancaster University, Lancaster, UK. The same measurement system and procedure were used in both locations. During the measurement intervals, which lasted ∼30 min, the participants were asked to keep their eyes open, without a fixation point.

A 16-channel system (V-Amp, Brain Products, Germany) was used to record the EEG at 1 kHz. An 8 source/8 detector LED system (NIRScout, NIRx, Germany) was used to record the fNIRS at 31.25 Hz. Locations of the resulting 16 EEG electrodes and 11 fNIRS sensors are shown in Fig. 1C. In addition, the EEG reference electrode was placed at FCz and the ground electrode at AFz. fNIRS measured relative changes in both oxygenated haemoglobin (oxyHb) and deoxygenated haemoglobin (deoxyHb) concentrations, and the oxyHb signal was used for this analysis: for the rest of the paper, it is the oxyHb signal that we are referring to when saying fNIRS signal or brain oxygenation time-series. Hence, oxygenation power refers to the power of the fNIRS time-series. An electrocardiogram (ECG) recorded the heart rate with bipolar precordial lead and a sampling frequency of 1.2 kHz, enabling sharp R-peaks to be reliably detected. The electrodes were placed on each shoulder and over the lower left rib, in a similar position as the D2 lead electrodes. A respiration belt wrapped around the participant’s chest measured the respiration effort (Biopac TSD201 Respiratory Effort Transducer, Biopac Systems Inc., CA, USA), also with a sampling frequency of 1.2 kHz. Both ECG and respiration effort were measured with a signal conditioning system with 24-bit A/D conversion (CardioSignal, Institute Jožef Stefan, Slovenia). Figure 1D shows examples of time-series measured from a member of the control group.

### Data preparation

All analysis was done using MATLAB, and the toolbox MODA was employed for the time-frequency analysis.^32^ First, continuous 25 min time-series were extracted from the data. For each participant, the same 25 min interval was used for the different types of time-series, as simultaneous recordings were needed for the coherence analysis. For the different time-series, before power and coherence analysis, the data were detrended by subtracting a best-fit third-order polynomial. The data were also bandpass filtered between 0.005 and 2 Hz with a zero-phase Butterworth bandpass filter. Respiration and ECG time-series were resampled to 100 Hz for peak detection analysis. The EEG was resampled to 31.25 Hz to match the fNIRS sampling frequency for fNIRS-EEG coherence analysis. The IHR, instantaneous respiration rate (IRR) and fNIRS time-series were resampled to 20 Hz for coherence analysis. An overview of the analysis methods and parameters can be found in Table 2. Non-linear mode decomposition was used to remove the cardiac artefact in EEG caused by cross-talk between brain electrical activity and heart electrical activity.^33^

**Table 2:** Summary of analysis methods and parameters

Analysis	Method	Parameter	*N*
Heart rate	Peak detection and ridge extraction	WT: f_0_ = 2 f ∈ [0.6,2] f_s_ = 100 Hz	15 Ad, 18 C
Respiration rate	Peak detection and ridge extraction	WT: f_0_ = 1 f ∈ [0.1,0.6] f_s_ = 100 Hz	19 Ad, 14 C
IHR power	Time-averaged WT	WT: f_0_ = 1 f ∈ [0.005,2] f_s_ = 20 Hz	15 Ad, 18 C
IRR power	Time-averaged WT	WT: f_0_ = 1 f ∈ [0.005,2] f_s_ = 20 Hz	19 Ad, 14 C
fNIRS power and coherence	Time-averaged WT and WPC	WT: f_0_ = 1 f ∈ [0.005,4] f_s_ = 31.25 Hz	19 Ad, 20 C
IHR-respiration coherence	WPC	WT: f_0_ = 1 f ∈ [0.005,2] f_s_ = 20 Hz	15 Ad, 14 C
IHR-fNIRS coherence	WPC	WT: f_0_ = 1 f ∈ [0.005,4] f_s_ = 20 Hz	15 Ad, 18 C
Respiration-fNIRS coherence	WPC	WT: f_0_ = 1 f ∈ [0.005,4] f_s_ = 20 Hz	19 Ad, 14 C
fNIRS-EEG coherence	WPC	WT: f_0_ = 1 f ∈ [0.005,4] f_s_ = 31.25 Hz	19 Ad, 20 C
EEG power and coherence	Time-averaged WT and WPC	WT: f_0_ = 1 f ∈ [0.005,4] f_s_ = 31.25 Hz WFT: f ∈ [4,48] f_s_ = 142 Hz	19 Ad, 20 C

N, number (of participants); AD, Alzheimer’s disease group; C, control group; WT, wavelet transform; WFT, windowed Fourier transform; WPC, wavelet phase coherence; IHR, instantaneous heart rate; IRR, instantaneous respiration rate; f_s_, sampling frequency; f_0_, frequency resolution parameter.

### Peak detection

To detect the IHR and IRR, peak detection was used and then checked using ridge extraction.^34^ A custom MATLAB script identified the R-peaks in the ECG or the maxima in the respiration effort signal. At the mid-point between two peaks the IHR or IRR was set to the inverse of the time between the peaks. Linear interpolation was then used to find the values between each of these points and produce equidistantly sampled time-series. The resulting time-series of IRR or IHR had a sampling frequency of 100 Hz. However, by construction, these time-series cannot contain information on frequencies higher than half of the heart rate or respiration rate frequencies. Hence, they were downsampled to 20 Hz before further analysis. The average heart/respiration rate was found as the average of the IHR/IRR. To measure the variability in the heart and respiration rates, the standard deviations (SDs) of the IHR and IRR were found. When we refer to the respiration signal throughout the manuscript, we refer to the respiration effort, i.e. the raw respiration time-series as seen in Fig. 1D.

### Physiological oscillations: frequency bands

Previous time-frequency analysis of time-series such as blood flow and cardiac function has shown that cardiovascular oscillations are manifested in specific frequency intervals, corresponding to different physiological processes.^21^ These frequency intervals and their physiological origins are summarized in Table 3, and range from 0.005 to 2 Hz. The cardiovascular oscillations overlap with slow oscillations in the EEG,^35^ whose origin is still debated.

**Table 3:** Frequency bands

	Frequency (Hz)	Period (s)	Physiological process
Endothelial	0.005–0.0095	105–200	Endothelial activity that modulates the activity of SMCs by the release of substances other than NO.
Endothelial NO	0.0095–0.021	48–105	Endothelial activity that modulates the activity of SMCs by the release of NO.
Neurogenic	0.021–0.052	19–48	Neurogenic activity by the autonomous nervous system that modulates the activity of SMCs by the release of substances.
Myogenic	0.052–0.145	7–19	SMCs alter their activity in response to intravascular pressure changes, and thus contract or relax.
Respiration	0.145–0.6	1.7–7	Respiration activity.
Cardiac	0.6–1.7	1.7–0.6	Heart activity.

Cardiovascular frequency bands with frequency ranges, the corresponding periods in seconds and the underlying physiological process.^21^

The lowest frequency of interest was therefore 0.005 Hz. We analysed fNIRS and EEG up to 4 Hz to include the δ band in EEG. fNIRS is not thought to contain oscillatory modes faster than the cardiac activity.

Power and coherence, which are explained below, were averaged within the frequency bands to obtain a single value per person per probe or probe combination.

### Strength of oscillations: wavelet transform

We are interested in the presence and strength of the cardiovascular oscillations, and in whether there are differences between the Alzheimer’s disease and control groups. Previous time-frequency analysis of cardiovascular oscillations has shown that these oscillations are time-varying.^21^ This is likely due to biological systems being open systems, with interacting sub-systems (e.g. cardiorespiratory interactions) that are also interacting with the environment (e.g. through breathing, the body inhales oxygen from the environment and exhales carbon dioxide). This time-variability is why time-frequency analysis can be more appropriate than frequency-domain analysis when studying cardiovascular oscillations. In addition, cardiovascular oscillations are manifested in the frequency interval 0.005–2 Hz, meaning that the oscillations have periods from around 0.5 to 200 s. To capture such multi-scale dynamics, a logarithmic frequency resolution is desirable. The WT is therefore an excellent choice for the analysis,^36^ and can be thought of as a convolution of the time-series with a mother wavelet. The wavelet is finite and centred on one time, so to achieve time resolution the wavelet is moved across the time-series. In order to investigate various frequencies, the wavelet is stretched and squeezed. The Morlet wavelet was used in this analysis, with a frequency resolution parameter of 1.

From the transform, an instantaneous amplitude and an instantaneous phase for each frequency are obtained. We average the amplitude over time, and square it, to find the time-averaged WT power. This is similar to the use of a Fourier spectrum, but has the advantages discussed above.

### Coordination of oscillations: wavelet phase coherence

In order to quantify potential interactions between sub-systems of the body, we treat the cardiovascular system and the brain as interacting phase oscillators.^21^ If the phase difference between two oscillations is constant throughout time, it indicates an underlying interaction, and we quantify the consistency of the phase difference using WPC.^25-27^ The instantaneous phase obtained from the WT, at a time *t* and frequency *k* for oscillator 1 is $\theta_{t,k}^{1}$ The phase difference between oscillator 1 and 2 is given by:

$$\Delta \theta_{t,k}=\theta_{t,k}^{1}-\;\theta_{t,k}^{2}.$$

The time-averaged WPC is then found as:

$$\mathrm{WP}C_{k}=\sqrt{{\left\langle \cos(\Delta \theta_{k})\right\rangle }^{2}+{\left\langle \sin(\Delta \theta_{k})\right\rangle }^{2}}.$$

The brackets indicate that the cosine and sine terms are averaged over all times. $\mathrm{WP}C_{k}$ takes values from 0 to 1, where 1 indicates a constant phase difference throughout the length of the time-series. There is one coherence value per frequency and, due to the properties of the WT discussed above, the WPC is suitable to investigate coherence in time-series that contain several oscillations of different frequencies. It is model-free, and completely independent of amplitude information. The WPC was calculated between fNIRS-fNIRS, fNIRS-IHR, fNIRS-respiration, fNIRS-EEG and IHR-respiration time-series.

We define the global coherence as the average coherence of all unique probe combinations in a frequency band, because the WPC between oscillator 1 and 2 is the same as the WPC between oscillator 2 and 1. For example, oxyHb global myogenic coherence is the average coherence in the myogenic band of 55 fNIRS probe combinations.

### Effect size

Effect size was calculated to quantify how different the groups are, when a significant difference was found. The effect size was calculated *post hoc* with a non-parametric adjustment to the Cohen’s *d*.^37,38^ Starting with the standard score *z*, found from ranks, *r* was calculated as

$$r=\;\frac{z}{N},$$

where *N* was the total sample size.^39^ Cohen’s *d* was then calculated as:

$$d=\;\frac{2r}{\sqrt{1-r^{2}}}.$$

An effect size between 0.5 and 0.8 is considered medium, while above 0.8 is considered large.

### Statistical analysis

The WPC computed between two randomly generated time-series is not necessarily 0, and so a method is needed for the detection of significant coherence. For this, inter-subject surrogates were used.^40^ This tests what coherence level can be obtained when calculating WPC between time-series from two different participants. This coherence can only be random, and cannot signify a coupling or interaction between the oscillations. So, for each participant, the significance threshold was chosen as the 95th percentile of the 166 inter-subject coherences at each frequency. The effective coherence was then found by subtracting this threshold from the original coherence, and only effective coherence is shown throughout the paper.

When testing for significant differences in power or effective coherence between the two groups the non-parametric two-sided Wilcoxon rank-sum test was used. *P*-values below 0.05 were considered as significant. Performing many significance tests increases the chance of false positives, known as the multiple comparison problem. In this analysis, coherence and power were averaged in time and frequency, and so only the number of sensor combinations contributes to the multiple comparison problem. Assuming all null-hypotheses were true (i.e. no difference between the groups), and α = 0.05, the number of expected null-hypotheses being incorrectly rejected was 2.75 for the fNIRS coherence analysis and 8.8 for the fNIRS-EEG coherence analysis. The probability of obtaining *X* or more positive findings from a total of *N* tries can be found from the binomial probability.^41^ The probability of a positive finding was set to 0.05, and the total number of tries was 55 for the NIRS coherence analysis and 176 for the NIRS-EEG coherence analysis. If the binomial probability of obtaining *X* positive findings was over 5%, the results were corrected for multiple comparison using the Benjamini–Hochberg (BH) correction.^42^

## Results

Here, we present results that were obtained from 25 min continuous recordings of fNIRS, EEG, ECG and respiration effort from the 39 participants (19 Alzheimer’s disease participants and 20 control participants), whose details are provided in Table 1. As reported in the previous section on participants, in some cases, the signals were not of sufficient quality for inclusion. The number of participants in each comparison is indicated in Table 2.

### Cardiorespiratory oscillations

**Figure 2:**
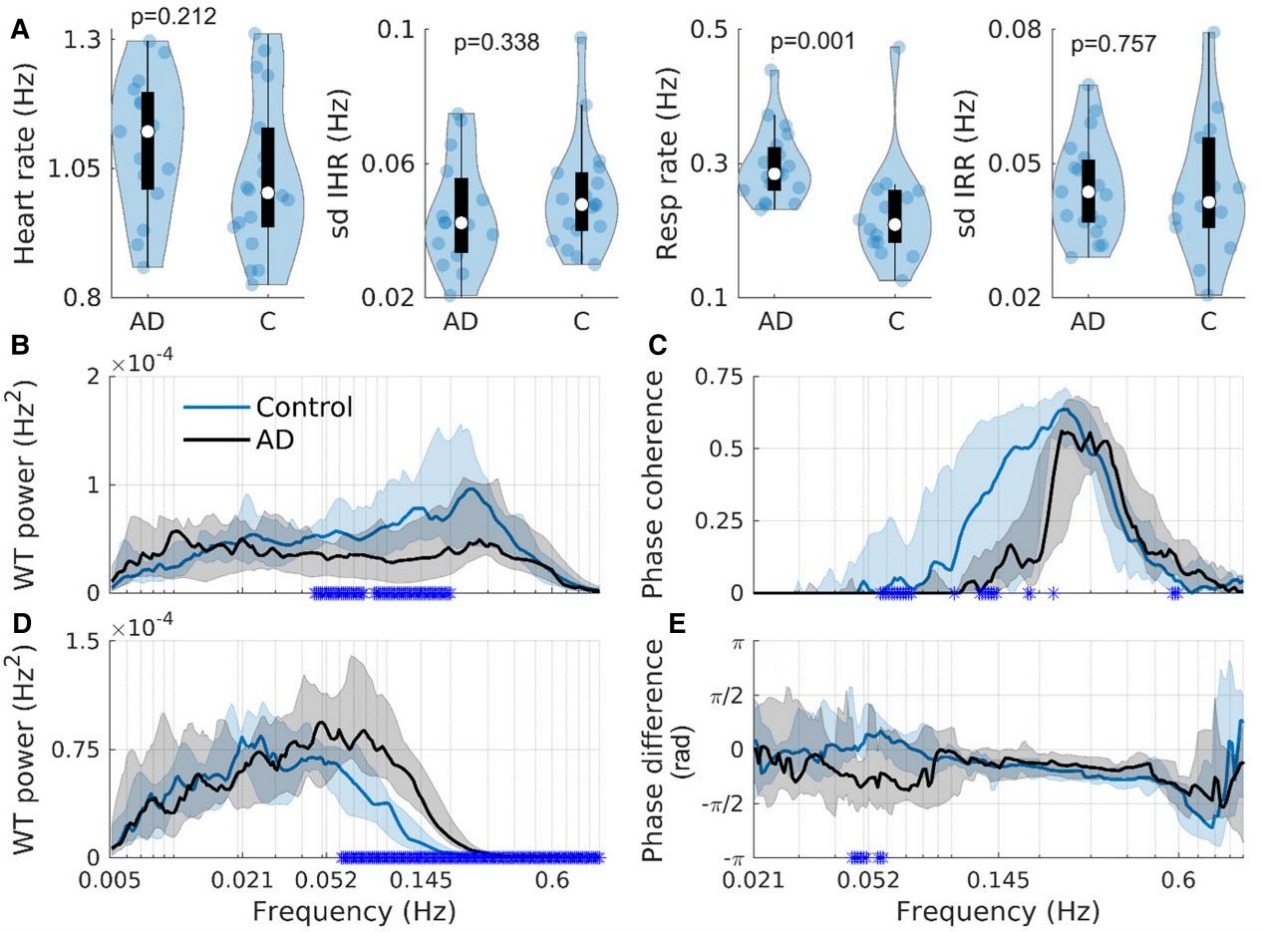
**Cardiorespiratory oscillations.** (**A**) Violin plots showing the average heart rate, SD IHR (N = 18 for controls, N = 15 for AD), average respiration rate and the SD IRR (N = 14 for controls, N = 19 for AD). Each point in the plots corresponds to one participant’s data. (**B**) The IHR WT power (N = 18 for controls, N = 15 for AD). (**C**) The WPC between the IHR and respiration time-series (N = 14 for controls, N = 15 for AD). (**D**) The IRR WT power (N = 14 for controls, N = 19 for AD). (**E**) The phase difference between the IHR and respiration time-series (N = 14 for controls, N = 19 for AD). A negative value indicates that respiration was preceding the IHR. The black and blue solid lines show the group medians, while the shaded areas show the 25–75th percentiles. A blue star on the *x*-axis indicates a significant difference (*P* ≤ 0.05) between the groups at that frequency. All *P*-values were calculated using the Wilcoxon rank-sum test. AD, Alzheimer’s disease; C, controls.

There are no significant differences in the average heart rate (*P* = 0.21) or SD of the IHR (*P* = 0.34) between the two groups (Fig. 2A). The average respiration rate is significantly different between the groups (*P* = 0.001), with an effect size of 1.45 (Table 4). The median rate is 0.21 Hz (∼13 breaths/min) for the control group, and 0.28 Hz (∼17 breaths/min) for the Alzheimer’s disease group (Fig. 2). The SD IRR is not significantly different (*P* = 0.76), see Fig. 2A. The control group has higher IHR power in the range 0.05–0.2 Hz (Fig. 2B), while the Alzheimer’s disease group has higher IRR power in the range 0.06–1 Hz (Fig. 2D).

**Table 4:** Effect size

	Cohen’s *d*
Average respiration rate	1.45
fNIRS power in neurogenic band	N10: 1.13, N11: 1.11
fNIRS power in myogenic band	N1: 0.89, N7: 0.85, N10: 1.16, N11: 1.19
fNIRS coherence in endothelial band	0.85
fNIRS coherence in neurogenic band	1.12
fNIRS coherence in myogenic band	1.22
fNIRS coherence in respiration band	0.88
fNIRS coherence in cardiac band	0.87
fNIRS-EEG coherence in myogenic band	0.76 ± 0.05
fNIRS-EEG coherence in neurogenic band	0.84 ± 0.16

The effect size calculations for various significant differences between the groups. The fNIRS power effect size is shown for each probe where a significant difference was found. For the fNIRS-EEG coherence in the myogenic band, the effect size is shown as mean ± SD of the 18 combinations with a significant difference.

The IHR-respiration effective phase coherence is significantly different between the two groups in the range 0.06–0.07 and ∼1.4 Hz (Fig. 2C), and the control group has higher coherence. In both groups, the phase difference is negative in the 0.145–0.5 Hz range, indicating that respiration is leading. The Supplementary material section 2 contains additional IHR results. Analysis of 25 min-recordings for all participants for which ECG was recorded (19 Alzheimer’s disease and 18 control) and 5 min-recordings from the originally included participants (15 Alzheimer’s disease and 18 control), where artefacts were avoided, show that the length of recording does not weaken the significant findings, but including the participants with abnormal ECGs does.

### Brain oxygenation oscillations

**Figure 3:**
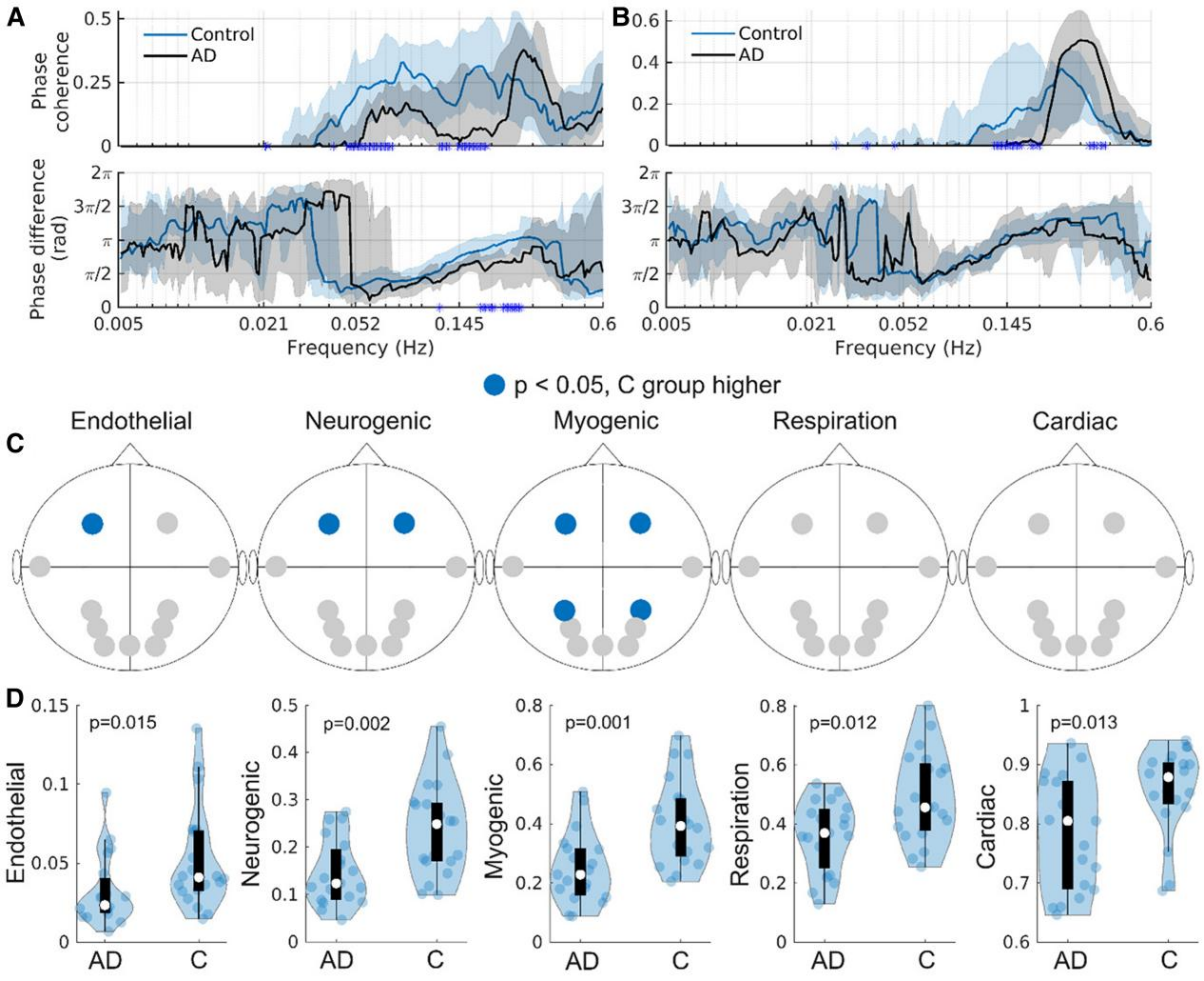
**Brain oxygenation oscillations.** (**A**) The WPC and phase difference between the IHR and brain oxygenation time-series at N11. N = 18 controls, N = 15 Ad. (**B**) The WPC and phase difference between respiration and brain oxygenation time-series at N11. N = 14 controls, N = 19 Ad. The black and blue solid lines show the group medians, while the shaded areas show the 25–75th percentiles. A blue star on the *x*-axis indicates a significant difference (*P* ≤ 0.05) between the groups at that frequency. (**C**) Significant differences (*P* ≤ 0.05) between the two groups in brain oxygenation power at specific probes, and in specific frequency bands, are indicated in blue. N = 20 controls, N = 19 Ad. (**D**) Violin plots showing the brain oxygenation global coherence in the five cardiovascular frequency bands. N = 20 controls, N = 19 Ad. All *P*-values were calculated using the Wilcoxon rank-sum test. AD, Alzheimer’s disease; C, controls. Each point in the plots corresponds to one participant’s data.

Next, phase coherence between respiration/IHR and fNIRS signals was found. In Fig. 3A and B, this is shown at location N11 (see Fig. 1C for sensor locations). The coherence with the remaining fNIRS sensors is shown in the Supplementary material, sections 3 and 4. For both groups, IHR-fNIRS effective phase coherence was seen in the 0.052–0.6 Hz frequency range. Respiration-fNIRS effective phase coherence is mostly found in the 0.145–0.6 Hz range. The phase difference increases steadily from ∼0.06 to ∼0.3 Hz, suggesting a consistent time lag between the two oscillators of around 2.5 s. In Fig. 3A, one can see that the IHR-fNIRS coherence is reduced in the Alzheimer’s disease group, which is consistent across all fNIRS channels. The phase difference is also significantly different between the two groups around 0.2 Hz, a result that is consistent in 9/11 fNIRS channels. The respiration-fNIRS coherence is lower in the control group in the 0.3–0.4 Hz range, which is consistent in 4/11 fNIRS channels. On the other hand, the coherence is lower in the Alzheimer’s disease group around 0.145 Hz, which is consistent in 7/11 fNIRS channels.

The Alzheimer’s disease group has reduced brain oxygenation power compared to the control group in the neurogenic and myogenic frequency bands, both at N10 and N11 (Fig. 3C). In the myogenic band reduced power is also seen at N1 and N7. In addition, the Alzheimer’s disease group has reduced power at N11 in the endothelial band. The probability of 1 or more positive findings, assuming all null hypothesis are true, is 45%, while for 2 or more it is 10%, and for 4 or more findings the probability is 0.16%. Further multiple comparison corrections are therefore not needed for the myogenic band, but have been applied to the neurogenic and endothelial bands. The significant difference in the endothelial band does not survive BH correction (original *P*-value 0.029, corrected *P*-value 0.21). The significant differences in the neurogenic band survive BH correction (original *P*-values 0.0035 and 0.0042, corrected *P*-values 0.0139 and 0.0139).

The global coherence in all frequency bands is significantly reduced in the Alzheimer’s disease group (Fig. 3D). Effect size calculations are shown in Table 4, and generally show large differences between the groups.

### Neuronal oscillations

For completeness, the results and discussion on neuronal oscillations extracted from the EEG are presented in the Supplementary material, section 6.

### Neurovascular oscillations

**Figure 4:**
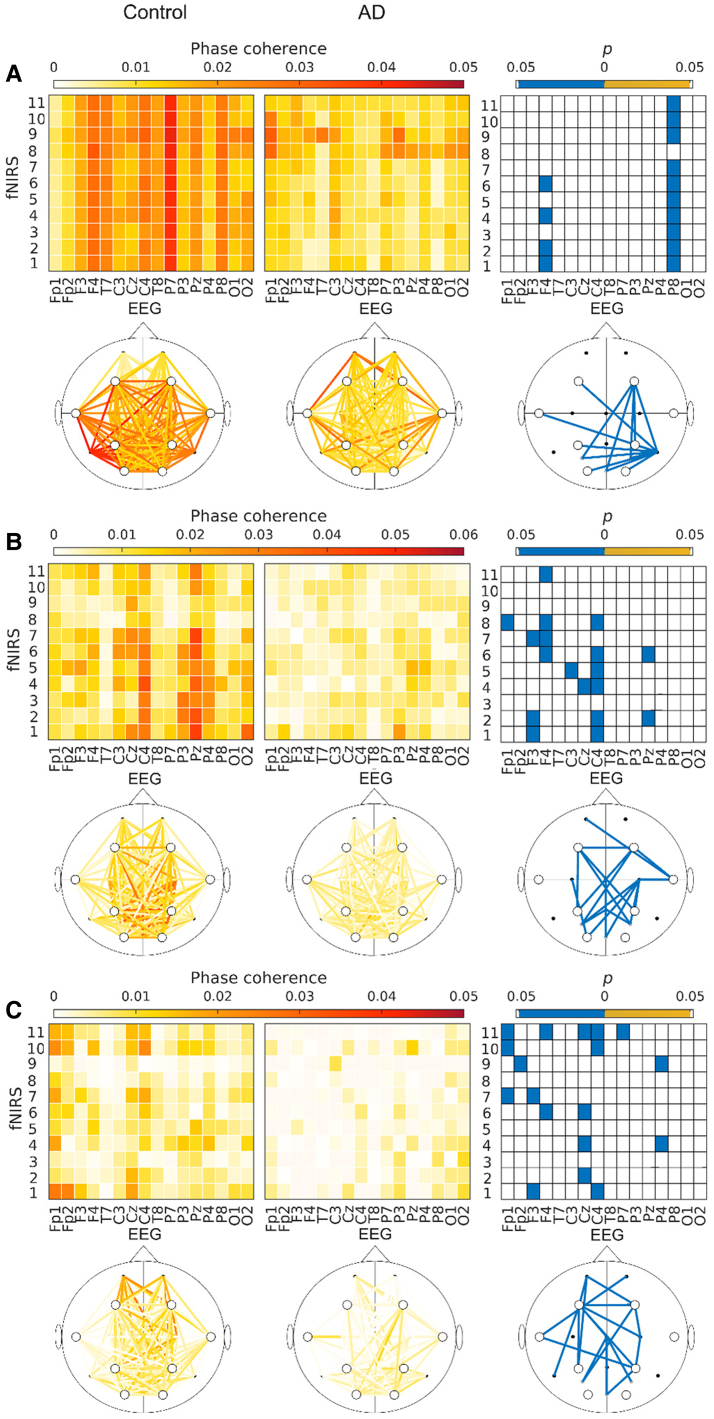
**Neurovascular oscillations.** (**A**) The WPC in the cardiac frequency band between fNIRS and electroencephalogram time-series, for all possible combinations. The left column shows the control group median, while the middle column shows the Alzheimer’s disease group median. The right column shows significant differences (*P* ≤ 0.05) between the groups. The square plots and head plots depict the same information. (**B**) As in (**A**), but for the myogenic band. (**C**) As in (**A**), but for the neurogenic band. All *P*-values were calculated using the Wilcoxon rank-sum test. AD, Alzheimer’s disease; C, controls. N = 20 controls, N = 19 Ad.

In the cardiac, myogenic and neurogenic frequency bands the Alzheimer’s disease group has reduced neurovascular coherence in 14/176, 18/176 and 18/178 of the fNIRS-EEG combinations, respectively (Fig. 4A–C). In the control group, the coherence in the myogenic frequency band is highest in the parietal and central electrodes, while in the Alzheimer’s disease group, it has more or less vanished (Fig. 4B). Coherence in the neurogenic band is generally low in both groups, but the control group has higher median values in the Fp1, Fp2 and central electrodes (Fig. 4C). The surrogate threshold at low frequencies is so high that the endothelial frequency band was not considered for the fNIRS-EEG coherence analysis. The probability of 14 or more positive findings, assuming all null hypothesis are true, is 5.9%, and therefore further multiple comparison corrections were needed in the cardiac band. None of the 14 combinations survives BH correction. The average effect size of the 18 significant combinations in the myogenic band is 0.76, while the average effect size in the neurogenic band is 0.84 (Table 4).

## Discussion

We analysed time-series of cardiovascular and neural origin, from Alzheimer’s disease patients and control participants in the resting-state, in an attempt to develop a non-invasive methodology for assessing the functioning of the NVU *in vivo*. We find that data from Alzheimer’s disease patients exhibit the following three main features:

- Decreased oxygenation power and decreased global oxygenation phase coherence compared to controls,
- Increased respiration rate compared to controls,
- Decreased neurovascular phase coherence compared to controls.

The following sections will discuss these findings and their implications.

### Vascular and neurovascular alterations in Alzheimer’s disease

We find stark changes in the oxygenation dynamics in the Alzheimer’s disease group compared to the control group, when considering either the power or the coordination of oscillations (Fig. 3C and D). The Alzheimer’s disease group has reduced fNIRS power at N10 and N11 in the myogenic and neurogenic frequency bands. The myogenic frequency band is linked to vasomotion caused by myogenic activity (i.e. SMCs activity),^21,43-45^ which has also been observed *in vivo* in the human cortex.^46^ The neurogenic frequency band is associated with vasomotion caused by nervous activity.^44,45^

The production of ATP requires oxygen, and so the brain relies on a sufficient supply of oxygen to meet its metabolic demand. ATP is essential for maintaining cellular pumps, which, in turn, facilitate vasomotion, thereby controlling blood flow and oxygen supply.^47^ Dysfunction in vasomotion is implicated in Alzheimer’s disease,^47^ such as hypercontractility.^48^ Disrupted vasomotion might contribute to cerebral hypoperfusion, which is observed in Alzheimer’s disease.^8^ Mild cerebral hypoperfusion can negatively affect neuronal function, through disrupted neuronal protein synthesis, which is important for synaptic plasticity.^49^ Hypoperfusion can also disrupt the ability of neurons to generate action potentials through decreased ATP production.

Another aspect of vasomotion especially relevant to Alzheimer’s disease is that vasomotion is thought to contribute to clearance of amyloid-beta from the brain.^50^ The reduced power might be a sign of reduced vasomotion and therefore reduced clearance in the Alzheimer’s disease brain. The reduced clearance might further affect the vasomotion, as amyloid-beta has a negative effect on endothelial cells.^51^ The need for additional energy in response to increased neuronal activity is signalled to the local microvasculature by neurons and astrocytes. This signal propagates further up the vascular tree, to the arteries, due to the electrical coupling between endothelial cells.^9^ The endothelial cells are also in electrical contact with SMCs^52^ and therefore reduced functioning of endothelial cells can impact vasomotion causing a vicious circle. The control of cerebral blood flow is orchestrated by the NVU.^9^ Information flow is needed between the various cell types, such as neurons, astrocytes, pericytes, SMCs and endothelial cells. By calculating the phase coherence between oxygenation and neural signals, we aim to quantify this information flow, and thereby find a marker of the efficiency of the NVU.^17^ We found previously that neurovascular phase coherence decreases with age and with Huntington’s disease,^17,53^ and we now show that this coherence is also reduced in Alzheimer’s disease. This indicates reduced cooperation between cells in the NVU or, correspondingly, reduced neurovascular coupling.

Patients with Alzheimer’s disease have reduced microvascular density compared to controls.^6^ Further, morphological changes like kinking and focal constriction of the microvasculature have been seen in the Alzheimer’s disease brain,^51^ as well as a thickening of the basement membrane in Alzheimer’s disease participants.^51^ The morphological changes and thickening of the basement membrane could indicate that the cardiac pulse is not propagated to the microvasculature as efficiently as in control participants. These differences might explain why the cardiac coherence, and the coherence in the other frequency bands, are lower in the Alzheimer’s disease group. In addition, loss of neurons and altered functional connectivity could also cause the brain oxygenation to be less coherent.

Altered oxygen dynamics in the brain might also be linked to systemic cardiovascular oscillations,^54^ as the blood is oxygenated in the lungs and pumped by the heart. We find that the Alzheimer’s disease group has a higher average respiration rate compared to the control group. Respiratory dysfunction is observed in Alzheimer’s disease, such as decreased respiratory muscle strength compared to healthy controls.^55^ A previous study found that a higher respiration rate was linked to cognitive impairment.^56^ Another study found that both Alzheimer’s disease and controls had similar breathing rates of around 18 breaths/min.^57^ Further investigations are needed to confirm whether or not a higher respiration rate is common in Alzheimer’s disease patients.

The reduced IHR-oxygenation coherence in the lower respiratory band (∼0.145–0.2 Hz) seen in the Alzheimer’s disease group (Fig. 3; Supplementary material section 3) is likely due to their increased respiration rate. Across frontal, parietal and occipital locations the Alzheimer’s disease group has reduced coherence in the myogenic frequency range, indicating a systemic origin. The reduction could be linked to disrupted vasomotion and the non-efficient propagation of the cardiac pulse discussed above. In both the control and Alzheimer’s disease groups the IHR is leading the respiration oscillation in the myogenic band, indicating that the myogenic oscillation is propagated to the brain, as the SMCs responds to pressure changes caused by the cardiac pulse.

### Concurrent EEG, fNIRS, ECG and respiration combined with state-of-the-art time-frequency analysis as a potential biomarker

In addition to contributing to the understanding of physiological changes in Alzheimer’s disease, a secondary goal of the analysis was to consider the potential of these methods to create a biomarker. In the research setting, Alzheimer’s disease can be defined using the National Institute on Aging and Alzheimer’s Association Research framework.^58^ This is a purely biological definition (i.e. not dependent on clinical symptoms), in which Alzheimer’s disease is identified through Amyloid-beta, Tau and Neurodegeneration (A, T and N). These can be assessed by employing CSF, PET and MRI.^58^ Functional MRI resting-state networks and blood-based tests also have potential as biomarkers.^59^ Most of the mentioned biomarkers are expensive and some are invasive. As Alzheimer’s disease is a global disease, having access to less-expensive, non-invasive and also portable techniques is desirable. In this respect, EEG and fNIRS are potential candidates.^60,61^ If either EEG or fNIRS is taken in isolation, one obtains only an incomplete picture as neuronal activity and oxygenation are interlinked, and dependent on cardiorespiratory function. Fortunately, as is done in the present study and many others,^62^ EEG and fNIRS can be combined in a single scan. Previous work related to Alzheimer’s disease has been performed by Perpetuini *et al*.^63^ and Chiarelli *et al*.,^64^ where differences between the Alzheimer’s disease group and control group were found in both studies. We now show that the inclusion of simultaneous measurements of ECG and respiration enables metabolic aspects to be taken into account.

Previous studies employing fNIRS to study oxygenation dynamics in dementia have generally focused on absolute values, Pearson correlation and entropy.^61,65^ In contrast, in the present work, we have used a non-autonomous oscillatory dynamics approach.^66^ Combining non-invasive measurements of neurovascular function with time-frequency analysis methods—specifically selected to study oscillations with time-varying frequencies—we have revealed clear differences between the Alzheimer’s disease and control groups. Also focusing on time-dependence, Cruzat *et al*.^67^ showed that the temporal irreversibility of brain activity measured by fMRI and EEG is reduced in Alzheimer’s disease patients. They argue that this indicates that the unhealthy brain has activity closer to equilibrium dynamics. This might mean that the brain is less able to respond to environmental demands, or that intrinsic couplings are reduced. Non-autonomous dynamical systems^68^ (i.e. systems with explicit time-dependence of their characteristic frequencies and couplings) have inherent time-irreversibility. While both approaches consider time-dependence, a strength of focusing specifically on oscillations is the clearer link to physiology.^69,70^ Thus, our finding of reduced myogenic neurovascular coherence can be linked to decreased efficiency of the NVU. As such, our results provide a potential avenue for *in vivo* assessment of the efficiency of the NVU in the human brain.

### Limitations and strengths

This study has several limitations, such as a relatively small sample size. A strength of the study is that the Alzheimer’s disease patients were confirmed by standard biomarkers. The long recordings contain more information than shorter recordings and mean that sporadic or random changes in the investigated parameters would average out over time, making the significant differences between groups robust. However, it can be challenging for the participants to remain still during the recording. While some fidgeting can have impacted the measurements, it is very unlikely that this behaviour was oscillatory or that it can explain the group differences. This is illustrated by considering shorter time-series to repeat the analysis in Supplementary Figs. S3 and S6. To maintain alertness, the participants were asked to keep their eyes open during the measurement. However, we did not record electrooculography (EOG) signals or use EOG artefact rejection for the EEG time-series. Such an approach would not in fact have been feasible because time-series must be continuous for the coherence analysis of slow oscillations. This artefact affects only the EEG time-series by introducing large amplitude changes. As the WPC is independent of amplitude information, the artefact is unlikely to have affected the fNIRS-EEG coherence.

fNIRS measures only the relative changes in oxygenated and deoxygenated haemoglobin concentrations, and the exact locations of these changes are often unclear. This uncertainty complicates the quantification of absolute changes in these concentrations.^71^ We use wavelengths of 760 and 850 nm, thus avoiding wavelengths in the 770–800 nm range previously discussed as giving less accurate measurements.^71^ Haemodynamic changes in the scalp likely affect the amplitude of the signal more than the phase. However, amplitude-based measures are prone to cross-talk, movement artefacts, and other noise sources. For this reason, our approach emphasizes phase, as phase coherence has been shown to be more resilient to various types of noise.^27^ Additionally, fNIRS is a relatively inexpensive, portable and non-invasive method,^72^ making it a promising tool for monitoring haemodynamics in dementia.

Nested data is common in neuroscience and requires particular attention in their statistical evaluation.^73^ Here, we aggregate data so that for each statistical test only one value per participant is considered. We check for group differences at the probe level for spectral power and at the global level for fNIRS coherence. We also apply inter-subject surrogates to ensure that coherence is statistically significant.

### Concluding remarks

This work shows clear evidence of changes in brain oxygenation dynamics in Alzheimer’s disease, thereby supporting suggestions that vascular changes contribute to neurodegeneration. We detected reduced oxygenation power in the neurogenic and myogenic frequency bands, which are both associated with vasomotion. Reduced vasomotion can contribute to insufficient delivery of oxygen and nutrients and to reduced clearance of amyloid-beta which is known to aggregate in Alzheimer’s disease. Not surprisingly, we see direct evidence of reduced efficiency of the NVU in Alzheimer’s disease, investigated by phase coherence between EEG and fNIRS. Another important discovery is the significant increase in the frequency of respiration in participants with Alzheimer’s disease, suggesting the presence of inflammation.

The present work stands on two pillars. First, we consider both the neuronal and the cardiovascular aspects of brain function to understand changes in Alzheimer’s disease. Second, we apply multi-scale oscillatory dynamics analysis to gain neurophysiological insight into these changes.

After further validation, such a method could be used for the evaluation of treatments and routine follow-ups. With disappointing results from protein-focused drug trials, the vasculature and NVU are promising targets for future treatments of Alzheimer’s disease.

## Supplementary Material

fcaf007_Supplementary_Data

## Data Availability

The data will be made available on Lancaster University PURE depository, accessible for researchers that share their re-use intention (10.17635/lancaster/researchdata/692). The toolbox MODA is freely available on GitHub: https://github.com/luphysics/MODA. It was developed by the Nonlinear & Biomedical Physics Group at Lancaster University and the Nonlinear Dynamics and Synergetic Group at the University of Ljubljana.
