# Supplementary material for: Neurovascular phase coherence is altered in Alzheimer’s disease
Source: Brain Commun. 2025 Feb 3;7(1):fcaf007. doi: 10.1093/braincomms/fcaf007 (PMC11852277; doi:10.1093/braincomms/fcaf007)
Supplement: fcaf007_Supplementary_Data [file fcaf007_Supplementary_Data.pdf]

Supplementary Material for "Neurovascular phase coherence is altered in Alzheimer's disease"

Juliane Bjerkan[1], Bernard Meglič[2], Gemma Lancaster[1], Jan Kobal[2], Peter V. E. McClintock[1], Trevor J. Crawford[3], Aneta Stefanovska[1,*]

[1]Department of Physics, Lancaster University, Lancaster, LA1 4YB, UK
[2] Department of Neurology, University Medical Centre, 1525 Ljubljana, Slovenia
[3] Department of Psychology, Lancaster University, Lancaster, LA1 4YF, UK
[*]Corresponding author

December 9, 2024

Contents

1 Introduction 1

2 ECG analysis 1

3 Instantaneous heart rate and oxygenation coherence 4

4 Respiration and oxygenation coherence 5

5 Reproducibility of oxygenation power 6

6 EEG analysis, results and discussion 6
6.1 Analysis . . . 6
6.2 Results . . . 6
6.3 Discussion . . . 8

1 Introduction

This document contains supplementary material for the paper "Neurovascular phase coherence is altered in Alzheimer's disease". Section 2 discusses how some apparently healthy subjects were excluded on the basis of their abnormal ECG patterns. In sections 3 and 4 the coherence between instantaneous heart rate and oxygenation, and between respiration and oxygenation are shown for all fNIRS probes. The reproducibility of oxygenation power is discussed in Section 5. Section 6 contains additional information about the analysis, results and discussion for the EEG data.

2 ECG analysis

Despite not being diagnosed with heart problems, 4 of the participants had abnormal ECG traces, as can be seen in Supplementary Figure 1. The participants in Supplementary Figure 1A,B had many instances of ectopic heart beats throughout the entirety of the ∼30 minutes recordings. The participant in Supplementary Figure 1C might have persistent atrial fibrillation, and the participant in Supplementary Figure 1D might have hyperkalemia. The instantaneous heart rates (IHRs) of all 4 participants were clearly atypical, with very large variability. In all cases there was a problem with the conductance of the heart. These participants were therefore excluded from IHR analysis as stated in the main manuscript.

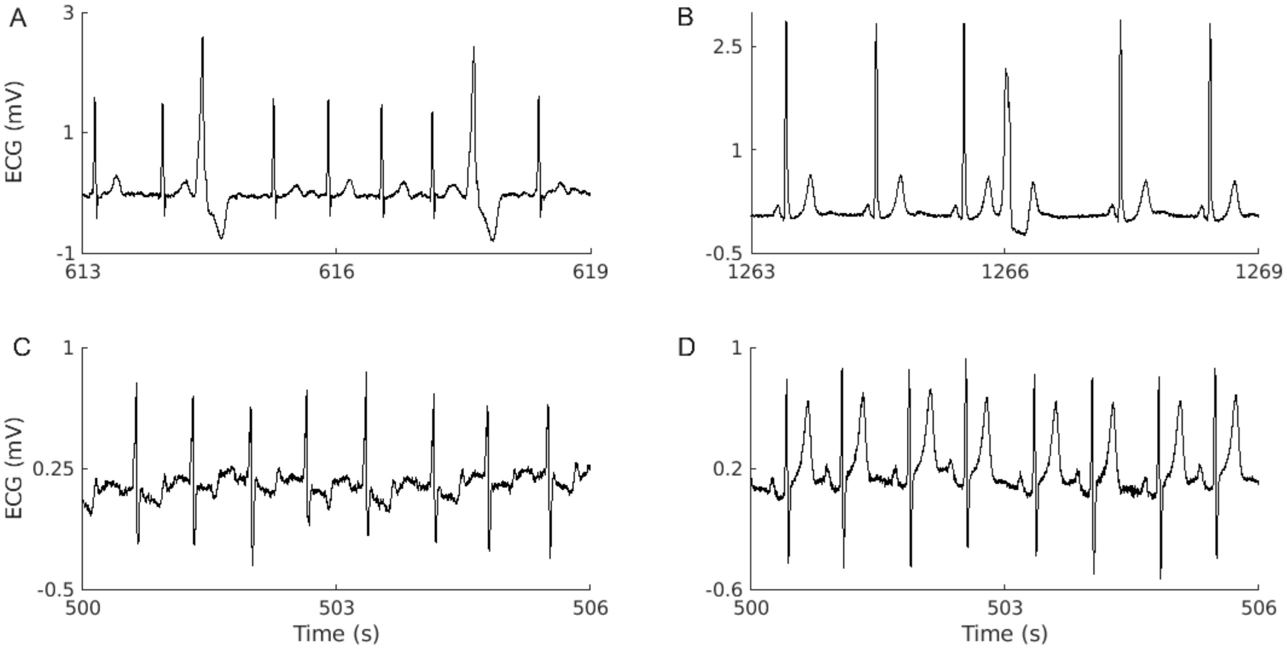

Supplementary Figure 1: Zoom-ins of the ECG recordings from (A) the control participant, and (B-D) AD participants that were excluded from the analysis due to abnormal ECGs. The recordings in (A) and (B) show ectopic heart beats, which were present throughout the length of the recordings. The recording in (C) does not have the characteristic ECG shape, potentially due to persistent atrial fibrillation. The recording in (D) have a large T-wave, in addition to a low S wave, potentially due to hyperkalemia. All 4 recordings resulted in abnormal instantaneous heart rates.

The results for IHR power and IHR–respiration coherence when these participants are included are shown in Supplementary Figures 2A and B respectively. The 75th percentile of IHR power is clearly affected by the additional 3 AD participants, and some of the significance in the 0.052-0.145 Hz range is now gone. On the other hand, the difference in IHR–respiration coherence is more significant, likely due to the increased statistical power when more people are included.

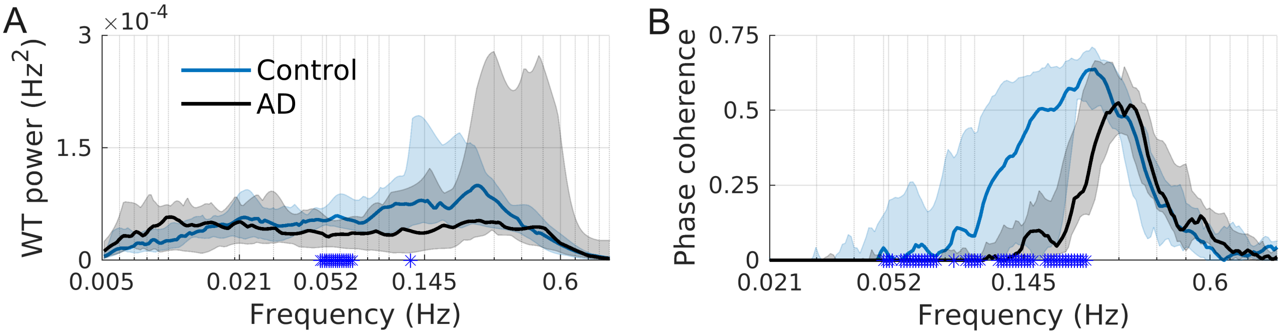

Supplementary Figure 2: (A) Instantaneous heart rate (IHR) power and (B) IHR–respiration coherence, with the excluded participants now included. The solid black and blue lines show the median group coherence, while the shaded areas show the 25–75th percentiles. Significant differences (p≤0.05) between the groups at particular frequencies, found using the Wilcoxon rank-sum test, are indicated by blue stars on the x-axis. AD = Alzheimer's disease, C = control group.) $N = 19$ for controls, $N = 18$ for AD.

Many studies of heart rate variability use 300 second recordings. We too calculated the IHR power for 300 seconds for the participants included in the main manuscript. Supplementary Figure 3 shows that the power is significantly reduced in the 0.052–0.2 Hz range, which is consistent with the results from the full recording. Shorter time-series limit the frequency interval which can be investigated at its lower end.

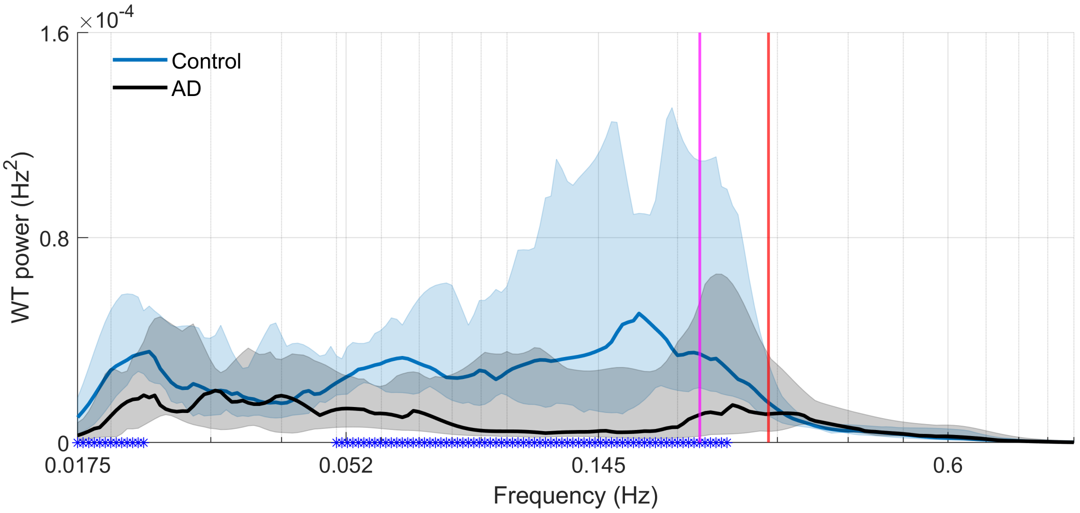

Supplementary Figure 3: Instantaneous heart rate power, calculated from 300 seconds time-series. The red vertical line shows the average respiration rate for the Alzheimer's disease group, while the green vertical line show the average respiration rate for the control group. The solid black and blue lines show the median group coherence, while the shaded areas show the 25–75th percentiles. Significant differences ($p \leq 0.05$) between the groups at particular frequencies, found using the Wilcoxon rank-sum test, are indicated by blue stars on the x-axis. AD = Alzheimer's disease, C = control group. $N = 18$ for controls, $N = 15$ for AD.

3 Instantaneous heart rate and oxygenation coherence

Supplementary Figure 4 plots the IHR-fNIRS coherence and phase difference for all 11 fNIRS channels.

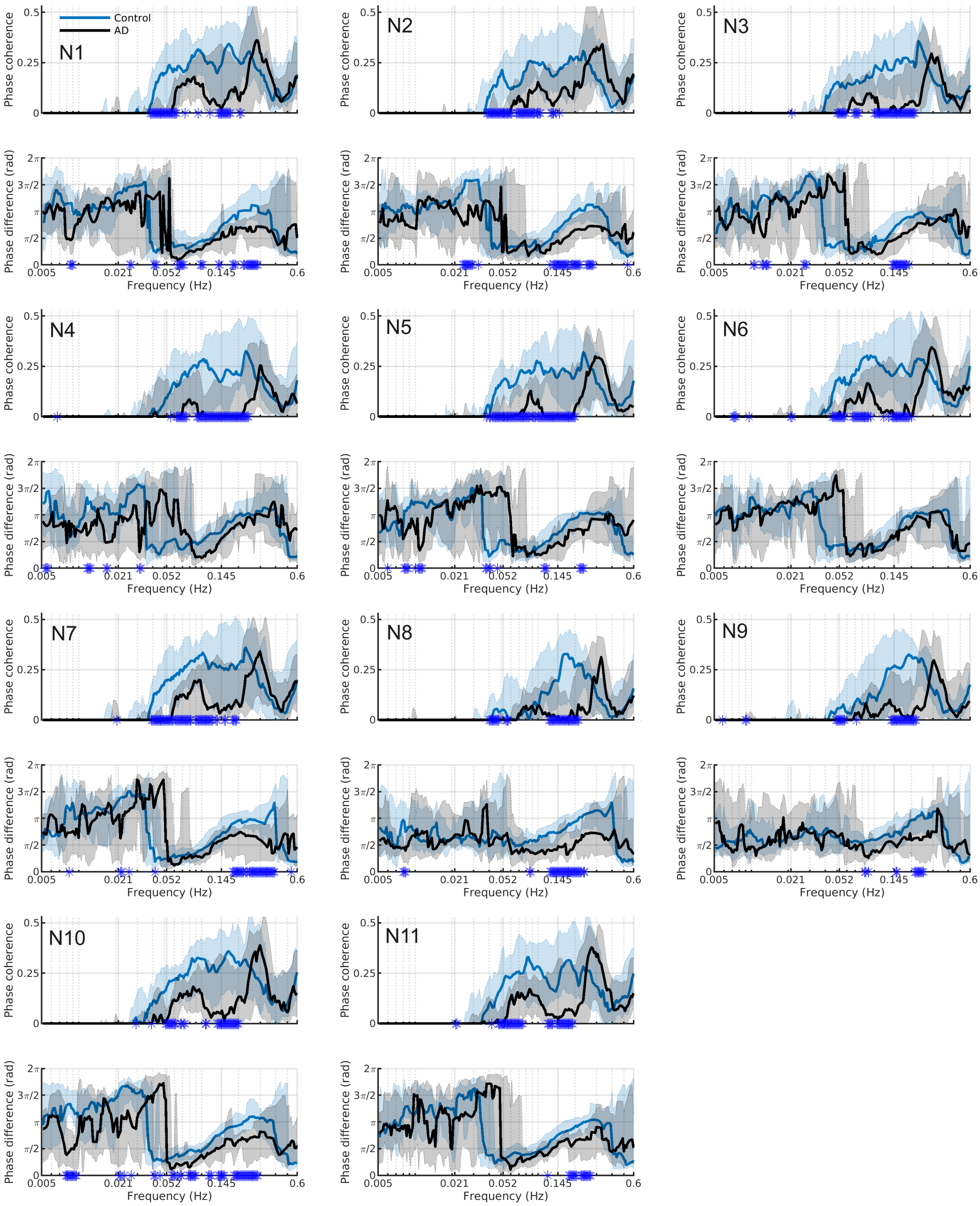

Supplementary Figure 4: Instantaneous heart rate (IHR)–fNIRS coherence and phase difference for all 11 fNIRS channels. The solid black and blue lines show the median group coherence, while the shaded areas show the 25–75th percentiles. Significant differences ($p \leq 0.05$), found using the Wilcoxon rank-sum test, between the groups at particular frequencies are indicated by blue stars on the x-axis. AD = Alzheimer's disease, C = control group. $N = 18$ for controls, $N = 15$ for AD.

4 Respiration and oxygenation coherence

Supplementary Figure 5 plots the Respiration–fNIRS coherence and phase difference for all 11 fNIRS channels.

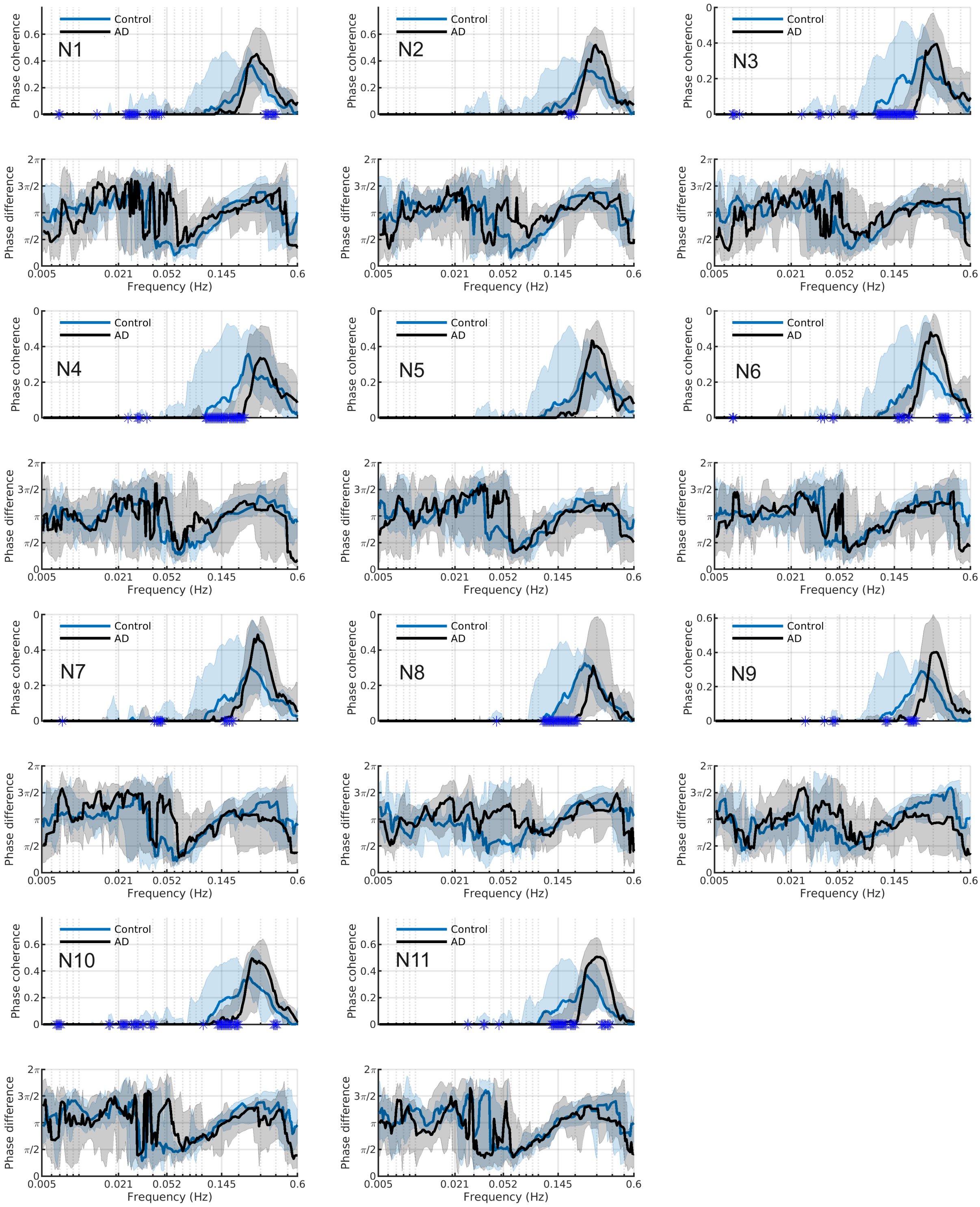

Supplementary Figure 5: Respiration–fNIRS coherence and phase difference for all 11 fNIRS channels. The solid black and blue lines show the median group coherence, while the shaded areas show the 25–75th percentiles. Significant differences ($p \leq 0.05$) between the groups at particular frequencies, found using the Wilcoxon rank-sum test, are indicated by blue stars on the x-axis. AD = Alzheimer's disease, C = control group. N = 14 for controls, N = 19 for AD.

5 Reproducibility of oxygenation power

To verify the reproducibility of our results, we have split the fNIRS time-series into two halves and calculated the wavelet power. The significant differences in power between the two groups for the two segments are shown in the figure below (Supplementary Figure 6). Combined with the IHR power calculated from 300 s (Supplementary Figure 3), this shows that the power results are consistent across segments.

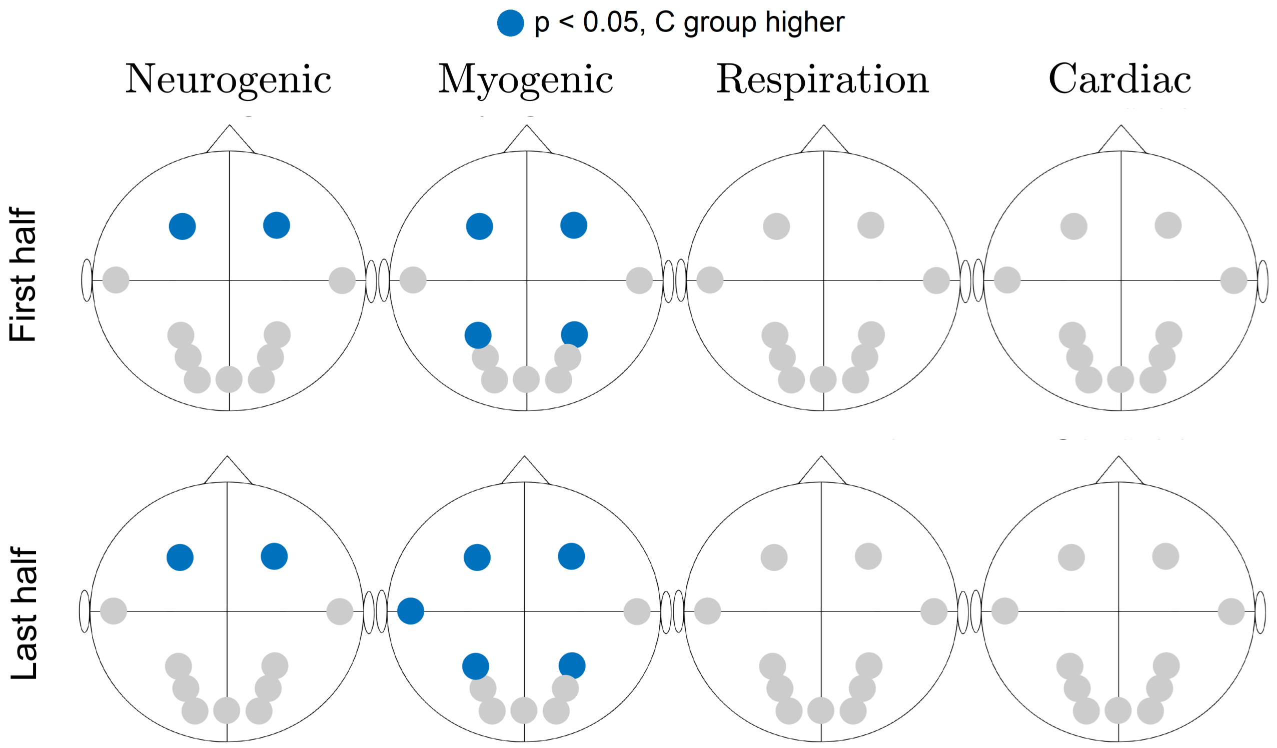

Supplementary Figure 6: Significant differences ($p \leq 0.05$) in fNIRS power between the Alzheimer's disease (AD) and control (C) groups, for fNIRS power calculated for the first and last 12.5 minutes. The Wilcoxon rank-sum test was used. It is evident that the results are consistent with those obtained from the whole-length (25 min) time-series as shown in the main part of the manuscript, Fig. 3. At location N9 there is an additional significant difference between the Alzheimer's disease and control groups in the last half of the recordings. The p-value is 0.35 for the first half and 0.07 for the whole-length. $N = 20$ for the C participants, $N = 19$ for AD patients.

6 EEG analysis, results and discussion

6.1 Analysis

The data acquisition and preparation is described in the main manuscript, as is the wavelet transform power, wavelet phase coherence and statistics. For frequencies up to the delta band the time-series were downsampled to 31.25 Hz, while for frequencies up to the gamma band the time-series were downsampled to 142 Hz. The wavelet transform was used to obtain power and phases for frequencies up to 4 Hz (delta band), while for frequencies up to the gamma band the windowed Fourier transform was used. The frequency bands used are myogenic (0.052-0.145 Hz), respiration (0.145-0.6 Hz), cardiac (0.6-1.7 Hz), delta (1.7-4 Hz), theta (4-7.5 Hz), alpha (7.5-14 Hz), beta (14-22 Hz) and gamma (22-48 Hz).

6.2 Results

The EEG power and phase coherence results are shown in Supplementary Figure 7. The AD group has higher power in the cardiac (2 channels), delta (3 channels), theta (9 channels) and gamma (5 channels) bands. The probability of 3 or more positive findings is 4%, and so the delta, theta and gamma results are significant. The

probability of 2 or more findings is 19%, and the cardiac result is therefore not significant.

For coherence significant differences are:

- AD group has significantly higher coherence in 11 combinations in the theta band, all of which includes the Cz electrode.
- The AD group has significantly higher coherence in 21 combinations (11 of which includes the Cz electrode) in the alpha band, while the control group has higher coherence in 8 combinations.
- The AD group has higher coherence in 15 combinations (8 of which includes the Cz electrode) in the beta, while the control group has higher coherence in 8 combinations.
- The AD group has higher coherence in 8 combinations in the gamma band, and the control group also has higher coherence in 7 combinations.

The probability of 11 significant findings is 3.8%, so the theta, alpha, beta and gamma results are significant. The myogenic and respiration results are not significant.

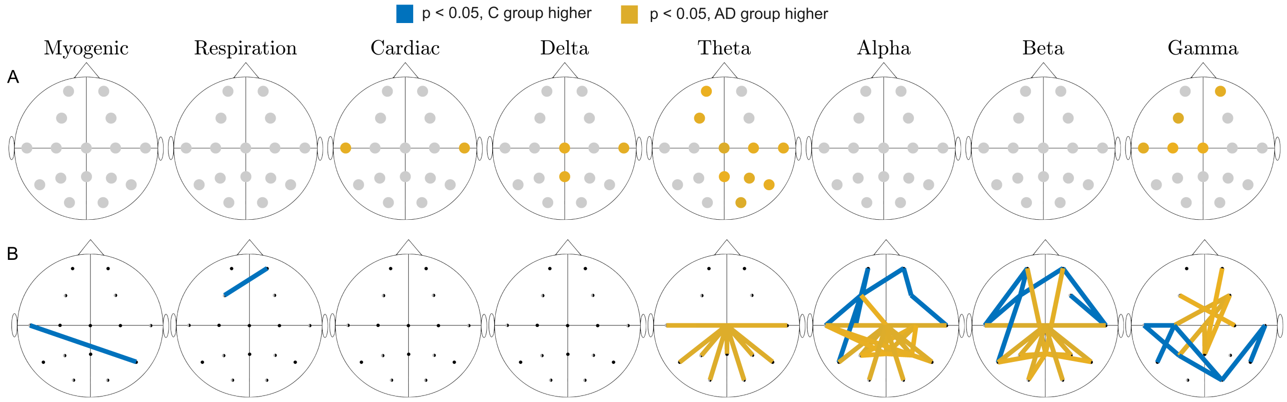

Supplementary Figure 7: (A) Significant differences ($p \leq 0.05$) in the EEG power in different frequency bands between the AD and C groups, found using the Wilcoxon rank-sum test. Orange (blue) circles indicate that the power was higher in the AD (control) group compared to the control (AD) group. Grey circles indicate there are no significant differences between the groups. (B) Significant differences ($p \leq 0.05$) in the EEG coherence in different frequency bands between the AD and C groups, found using the Wilcoxon rank-sum test. Orange (blue) lines indicate that the coherence was higher in the AD (control) group compared to the control (AD) group. AD = Alzheimer's disease, C = control. $N = 20$ for controls, $N = 19$ for AD.

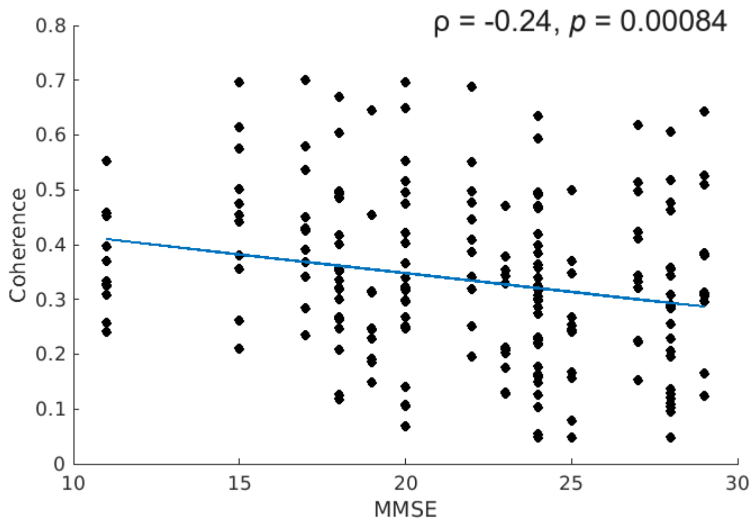

Supplementary Figure 8: Spearman's Rho between alpha coherence for the Cz probe with temporal, central, parietal and occipital probes (11 coherence values per person, shown as black dots) and the MMSE score, for participants with Alzheimer's disease. MMSE = mini mental state exam. The p-value is calculated from a permutation test. The blue line is the best fit linear function based on all data points.

6.3 Discussion

We show that both the power and coherence of EEG time-series are altered in AD, indicating altered neuronal activity. A slowing of the EEG is often found in AD, shown by increases in delta and theta power and decreases in alpha and beta power [1]. We find increases in delta and theta, but not decreases in alpha and beta power. However, our study was done with long, continuous recordings and eyes open, which is different to most EEG studies in AD. Alpha power is known to decrease in the eyes open condition, which might explain why we do not see a decrease [2, 3]. In addition, we see high variability in the alpha power in the AD group, which might also impact our ability to pick up the differences. With the sample sizes we can reliably pick up large effect sizes.

The increased alpha coherence in the central, temporal, parietal and occipital areas in the AD group is negatively correlated with disease stage as evaluated by the mini mental state exam score (Supplementary Figure 8). This could mean that increased coherence is associated with more atrophy, and that less synaptic connections could cause increased coherence. It would also mean that the volume conduction is more severe in the later stages of disease, and that causes increased coherence between EEG time-series from different probes.

Supplementary references

[1] Babiloni, C. *et al.* Abnormalities of cortical neural synchronization mechanisms in subjects with mild cognitive impairment due to Alzheimer's and Parkinson's diseases: An EEG study. *J. Alzheimer's. Dis.* **59**, 339–358 (2017).

[2] Kan, D. P. X., Croarkin, P. E., Phang, C. K. & Lee, P. F. EEG differences between eyes-closed and eyes-open conditions at the resting stage for euthymic participants. *Neurophysiology* **49**, 432–440 (2017).

[3] Stankovski, T., Ticcinelli, V., McClintock, P. V. E. & Stefanovska, A. Neural cross-frequency coupling functions. *Front. Syst. Neurosci.* **11**, 33 (2017).
